# Oleanolic acid rescues critical features of umbilical vein endothelial cells permanently affected by hyperglycemia

**DOI:** 10.3389/fendo.2023.1308606

**Published:** 2023-12-13

**Authors:** Javier Stelling-Férez, Ilaria Cappellacci, Assunta Pandolfi, José Antonio Gabaldón, Caterina Pipino, Francisco José Nicolás

**Affiliations:** ^1^ Department of Nutrition and Food Technology, Health Sciences PhD Program, Universidad Católica de Murcia (UCAM), Murcia, Spain; ^2^ Regeneration, Molecular Oncology, and TGF-β, IMIB-Pascual Parrilla, Hospital Clínico Universitario Virgen de la Arrixaca, Murcia, Spain; ^3^ Department of Medical, Oral and Biotechnological Sciences, StemTeCh Group, Center for Advanced Studies and Technology-CAST (ex CeSI-MeT), University G. D’Annunzio Chieti-Pescara, Chieti, Italy

**Keywords:** oleanolic acid, endothelial cells, angiogenesis, inflammation, chronic hyperglycemia, adhesion molecules

## Abstract

Skin wound healing is a physiological process that involves several cell types. Among them, endothelial cells are required for inflammation resolution and neo‐angiogenesis, both necessary for tissue restoration after injury. Primary human umbilical vein endothelial cells (C‐HUVECs) are derived from the umbilical cord. When women develop gestational diabetes, chronic exposure to hyperglycemia induces epigenetic modifications in these cells (GD‐HUVECs), leading to a permanent pro‐inflammatory phenotype and impaired angiogenesis in contrast to control cells. Oleanolic acid (OA) is a bioactive triterpenoid known for its epithelial cell migration promotion stimulation and higher tensile strength of wounds. However, the potentially anti‐inflammatory and pro‐angiogenic properties of OA are still under investigation. We tested OA on C‐ and GD‐HUVECs under inflammatory conditions induced by low levels of the inflammatory cytokine TNF-α. Reduced expression of adhesion molecules *VCAM1*, *ICAM1*, and *SELE* was obtained in OA‐pre‐treated C‐ and GD‐HUVECs. Additionally, protein VCAM1 levels were also decreased by OA. Coherently, monocyte adhesion assays showed that a lower number of monocytes adhered to GD‐HUVEC endothelium under OA pre‐treatment when compared to untreated ones. It is noteworthy that OA improved angiogenesis parameters in both phenotypes, being especially remarkable in the case of GD‐HUVECs, since OA strongly rescued their poor tube formation behavior. Moreover, endothelial cell migration was improved in C‐ and GD‐HUVECs in scratch assays, an effect that was further confirmed by focal adhesion (FA) remodeling, revealed by paxillin staining on immunocytochemistry assays. Altogether, these results suggest that OA could be an emergent wound healing agent due to its capacity to rescue endothelial malfunction caused by hyperglycemia.

## Introduction

During wound healing, epithelial cell migration is a crucial process to close and repair the skin barrier (1, 2). In pathological conditions, a physiologic impairment that halts wound healing may occur. Therefore, new treatments that could enhance or accelerate cell migration are currently of great interest. Oleanolic acid (OA), a bioactive triterpenoid present in a wide variety of plants, has shown promising effects on wound healing due to its cell migration activity on epithelial cells (3–7). Thus, OA activates epidermal growth factor receptor (EGFR), enabling a complex MAP kinase system, which in turn triggers c‐Jun phosphorylation and overexpression, a key transcription factor that enhances a gene expression cell migration program (7, 8). Besides these molecular effects, OA also changes elements of the epithelial cell architecture. OA promotes the assemble–disassemble turnover of focal adhesions (FAs) together with the actin and paxillin remodeling, a dynamic state that is evidence of cell migration (7, 9–11).

The findings of effects of OA on epithelial cells encouraged us to explore the role of this bioactive compound on other wound healing players, closely related to epithelial cells and their migration. In an acute wound, to reach wound closure, sequential phases must occur with the involvement of different cell types. The proliferative phase and the remodeling phase are critical for a correct wound resolution (12). These two stages need to take place after a correct inflammation mitigation in the wound, which is produced after skin injury and defines the inflammatory phase (13). At this point, in the injured blood vessels of the wound, endothelial cells respond by expressing adhesion molecules on the endothelium surface in order to facilitate the recruitment of immune cells to the wound site. In particular, they recruit immune cells that are known for their reparative properties, such as M2 macrophages and type T lymphocytes, which release anti‐inflammatory cytokines to modulate the wound inflammation milieu (14). The adequate resolution of this phase allows the wound to progress into the subsequent proliferative and remodeling phases, including angiogenesis and tissue regeneration. During angiogenesis, endothelial cells proliferate, migrate, and form a tube for the correct supply of nutrients, oxygen, and growth factors to the newly formed wound bed (15, 16). Briefly, angiogenesis is critical and occurs with cell migration during the proliferative and remodeling phases, supporting skin reepithelization.

However, when the wound is subjected to continuous inflammation, the other stages come to a halt, resulting in a chronic, non‐healing wound that may progress to an ulcer (17, 18). Indeed, there are many causes that trigger this condition, including trauma, burns, infections, or underlying chronic diseases such as diabetes (19). In fact, diabetes is one of the leading causes of impaired wound healing, and represents a complex issue due to its socioeconomic impact and the elevated number of patients (20, 21). For instance, one of its most severe complications is diabetic foot ulcer (DFU), in which the patient’s ulcer shows poor reepithelization and vascularization, leading to the amputation of the limb (20).

Diabetic ulcers display an excessive inflammatory response and deficient angiogenesis due to endothelium malfunction, which causes delayed healing and uncontrolled scar tissue formation (22–24). Thus, the use of *in vitro* cell models that can mimic endothelium diabetic features seems very relevant to studying possible strategies or agents that help rescue endothelial cells from this condition, and eventually restore their regular function. This is the case of human umbilical cord vein endothelial cells (HUVECs) exposed to hyperglycemia during pregnancy in mothers affected by gestational diabetes (GD) (25). Interestingly enough, this unique endothelial cell model (GD‐HUVECs) displays an altered phenotype that has been exhaustively studied and described (26). Although regular primary HUVECs are a well‐known *in vitro* model to study the process and molecular mechanisms related to inflammation and neo‐angiogenesis (27, 28), GD‐HUVECs are permanently damaged by hyperglycemia, thus showing a senescent pro‐inflammatory phenotype that leads to endothelial dysfunction (26). Therefore, GD‐HUVECs are a suitable model to study and to try to rescue an endothelium that is affected by diabetic ulcers and causes either a delay or even a halt on wound healing. Indeed, previous studies have shown that OA attenuates adhesion molecule overexpression under inflammation stimuli in C‐HUVECs (29, 30). Nevertheless, it might be very interesting to carry out these studies on GD‐HUVECs, which are endothelial cells experiencing a pathologic condition.

In this article, we have investigated the effects of OA on C-HUVECs and GD-HUVECs. Our results show that OA attenuates inflammatory responses, improves migration, and favors tube formation in both types of cells. Furthermore, these aspects are especially relevant in GD-HUVECs.

## Materials and methods

### HUVEC isolation and culture

All procedures adhered to the ethical standards of the Institutional Committee on Human Experimentation (reference number 1879/09COET) and to the principles of the Declaration of Helsinki. The protocol used was approved by the Institutional Review Board and informed consent was signed by every participating subject. Primary endothelial cells were collected from umbilical cord veins (HUVECs) of newborns delivered between the 36th and the 40th gestational week at the Hospital of Chieti and Pescara (Italy) from randomly selected Caucasian mothers affected by GD or not (control, C) following previously published methods (31). Briefly, veins of the umbilical cords were immediately collected after delivery, cannulated and perfused with 1 mg/mL collagenase 1A at 37°C. Obtained HUVECs were isolated in a base medium composed of DMEM/M199 (1:1) supplemented with 1% L‐glutamine, 1% penicillin/streptomycin, and 20% fetal bovine serum (FBS) (all from Biowest, Nuaillé, France). Then, the cell suspension was centrifuged at 1,200 rpm for 10 min, and the cell pellet was re-suspended in HUVEC base medium and plated on 1.5% gelatin‐coated (Sigma‐Aldrich, St Louis, MO, USA) tissue culture flasks. HUVECs were confirmed by the presence of specific markers such as von Willebrand factor, CD31 and CD34 positive, together with the induced expression of cell adhesion molecules ICAM1, VCAM1, and E‐selectin, and cytokines IL‐6 and IL‐8 under pro‐inflammatory stimuli, as well as the formation of cord‐like structures on Matrigel (25, 32). For all experiments, the cells were used *in vitro* between the 3rd and 5th passage, never exceeding the 5th passage. The HUVECs selected for the assays were grown on 1.5% gelatin‐coated tissue culture plates in HUVEC complete medium: low‐glucose (1 g/L) DMEM and M199 medium (ratio 1:1), supplemented with 10 μg/mL heparin (Sigma‐Aldrich, St Louis, MO, USA), 50 μg/mL endothelial cell growth factor (ECGF), 20% FBS, 1% penicillin/streptomycin, and 1% L‐glutamine. All experiments were performed, at least, in technical triplicate, using three different cellular strains (*n* = 3) of C‐ and GD‐HUVECs.

### Oleanolic acid preparation

OA (purity > 97%) (Merck, Darmstadt, Germany) was solubilized to a 25 mM final concentration in dimethyl sulfoxide (DMSO) (Sigma‐Aldrich, St Louis, MO, USA). Assay concentrations are indicated for each experiment in figure legends. MTT assays were performed in C‐ and GD‐HUVECs prior to functional assays, in order to optimize the OA/DMSO effect (see **Supplementary Figure 1**). In all the assays, DMSO concentration never exceeded 1% to avoid cytotoxic effects.

### RNA extraction and quantitative PCR

C‐ and GD‐HUVECs were seeded in 5-cm-diameter Petri dishes coated with 1.5% gelatin in HUVEC complete medium. When cells reached sub‐confluence (60%), cells were pre‐treated for 24 h with OA or DMSO (basal condition) in HUVEC complete medium with 10% FBS. After this, a 2-h serum‐starvation period was established in HUVEC serum‐starvation medium: low‐glucose (1 g/L) DMEM with 0.1% FBS, supplemented with 10 μg/mL heparin, 50 μg/mL endothelial cell growth factor (ECGF), 0.3% bovine serum albumin (BSA, from Sigma‐Aldrich, St Louis, MO, USA), 1% penicillin/streptomycin, and 1% L‐glutamine. After serum starvation, cells were treated with TNF‐α at 1 ng/mL, using this concentration for subsequent assays as well (28, 33, 34). Then, cells were incubated for 2, 6, and 24 h to induce the gene expression of adhesion molecules: vascular cell adhesion molecule 1, *VCAM1*, intercellular adhesion molecule 1, *ICAM1*, and, E‐selectin, *SELE*. At the times indicated above, RNA was extracted using the RNeasy‐mini system (Qiagen, Venlo, The Netherlands). Usually, 800 ng of RNA from independent samples was retro‐transcribed using iScript reagents (Bio‐Rad, Hercules, CA, USA). The obtained cDNA was used for quantitative PCR (qPCR) using the SYBR premix ex Taq kit (Takara Bio Europe/Clontech, Saint‐Germain‐en‐Laye, France) according to the manufacturer’s protocol. The primers used for the analyzed genes related to inflammation are indicated in **Table 1**. For gene expression analysis, qPCR cycles were normalized with glyceraldehyde 3‐phosphate dehydrogenase (*GAPDH*) gene expression according to the 2^−ΔΔCt^ method (35). The experiment was carried out on four different strains for C‐HUVECs and four different strains for GD‐HUVECs, each in technical triplicate. Analyzed data represent mean ± SEM.

**Table 1:** Different primers used to study the expression of several genes.

Gene name (GeneCards)/Primer name	Primer sequence 5’-3’
*GAPDH* Fwd	AGCTCAGGCCTCAAGACCTT
*GAPDH* Rev	AAGAAGATGCGGCTGACTGT
*ICAM1* Fwd	ACCATCTACAGCTTTCCG (Sigma KiCqStart)
*ICAM1* Rev	TCACACTTCACTGTCACC (Sigma KiCqStart)
*SELE* Fwd	GAGAATTCACCTACAAGTCC (Sigma KiCqStart)
*SELE* Rev	AGGCTTGAACATTTTACCAC (Sigma KiCqStart)
*VCAM1* (mix Fwd/Rev)	Proprietary sequence (Qiagen QuantiTect^®^) QT00018347

GADPH, glyceraldehyde-3-phosphate dehydrogenase; ICAM1, intercellular adhesion molecule 1; SELE, E‐Selectin; VCAM1, vascular cell adhesion molecule 1.

### MTT assay

The effects of increasing concentrations of OA on C‐HUVEC and GD‐HUVEC viability were assessed with the 3‐(4,5‐dimethylthiazolyl‐2)‐2, 5‐diphenyltetrazolium bromide (MTT, Sigma‐Aldrich) method (36). C‐ and GD‐HUVECs were seeded in 96‐well microplates, 2 × 10^4^ cells/cm^2^ (approximately 6500 cells per well), coated with 1.5% gelatin in HUVEC complete medium. When cells reached sub‐confluence (80%), a 24-h serum‐starvation period was established in HUVEC serum‐starvation medium (0.1% FBS). After this, cells were treated with vehicle control DMSO or OA, as indicated in **Supplementary Figure 1**, in 0.5% FBS media. After 24-h incubation, 20 µL of MTT 5 mg/mL in PBS was added to each well. Plates were incubated for 3 h at 37 °C and finally the absorbance at 540 nm was detected by a microplate reader (SpectraMAX 190, Molecular Devices, Sunnyvale, CA, USA).

### Western blot

C‐ and GD‐HUVECs were seeded in 5-cm-diameter Petri dishes coated with 1.5% gelatin in HUVEC complete medium. When cells reached sub-confluence (60%), cells were pre-treated for 24 h with OA or DMSO (basal) in HUVEC complete medium with 10% FBS. After this, a 2-h serum‐starvation period (0.1% FBS) was established in HUVEC serum‐starvation medium. Then, cells were treated with TNF‐α (1 ng/mL) for 1, 3, 6, or 24 h to induce inflammation. At the indicated times, cells were collected, washed twice with cold PBS, and lysed with 20 mM Tris, pH 7.5, 150 mM NaCl, 1 mM EDTA, 1.2 mM MgCl_2_, 0.5%, Nonidet p‐40, 1 mM DTT, 25 mM NaF, and 25 mM β‐glycerophosphate supplemented with phosphatase inhibitor cocktails (I and II) and protease inhibitors (all from Sigma‐Aldrich, St Louis, MO, USA). Total protein amount of all samples was measured and normalized by Bradford assay (37) (Sigma‐Aldrich, St Louis, MO, USA). Samples were analyzed by SDS‐PAGE followed by Western blot using the indicated antibodies (see the *Antibodies* section). Blots were revealed by using horseradish peroxidase substrate (ECL) (GE Healthcare, GE, Little Chalfont, United Kingdom) and images were taken with a ChemiDoc MP (Bio‐Rad, Hercules, CA, USA). To quantify Western blot protein bands, pictures in 8‐bit format were processed in ImageJ software. In every picture, a lane was established for each sample. In each lane, only the band with the specific size (kDa) of the protein of interest was quantified. For each total protein and its phosphorylated form, each band’s intensity peak was plotted, and subsequently, the area under the plot was measured by using “Wand tool” of ImageJ to finally obtain pixel intensity value. In order to normalize data, obtained intensity values were referred to those of the unphosphorylated form of the protein (total) or a loading control protein (β‐actin) if the unphosphorylated form was undetectable (non‐available antibody for the unphosphorylated form).

### Monocyte‐HUVEC adhesion assay

C‐ and GD‐HUVECs were seeded in six-well plates (200,000 cells/well) coated with 1.5% gelatin in HUVEC complete medium until they reached 60% confluence. At this time, cells were pre‐treated for 24 h with 20 µM OA in HUVEC complete medium with 10% FBS. When confluent, a 2-h serum‐starvation period was established washing and adding HUVEC serum starvation media. After this, cells were stimulated with TNF‐α (1 ng/mL) for 16 h. The U937 monocyte cell line (European Collection of Authenticated Cell Cultures, ECACC) was used to evaluate the adhesion to C‐ and GD‐HUVEC monolayers, as previously described (28, 33, 34). Briefly, the medium was removed from each HUVEC well, cells were gently washed with DMEM, and a suspension with 1 million monocytes was added to each well. Plates were incubated for 20 min, with gentle shaking at room temperature. Finally, to remove non‐adhered monocytes, HUVECs were gently washed and fixed with 1% paraformaldehyde. To identify the number of adherent monocytes for each tested strain, 12 counts were performed for every experimental condition (by using at least three different randomly selected high‐power fields, at 10× magnification) using Paula Nuc microscope (Leica Microsystems, Wetzlar, Germany). Images were acquired by using Paula software version 1.2.2. For this experiment, four different strains of both C‐HUVECs and GD‐HUVECs were used.

### Matrigel tube formation assay

C‐ and GD‐HUVECs were seeded on 12‐well plates coated with growth factor‐reduced basement membrane matrix gel, known as Matrigel (BD Biosciences, Franklin Lakes, NJ, USA) all in 10% FBS HUVEC complete medium. A number of 1.4 × 10^5^ cells/well was the suitable amount for the assay. After plating, cells were incubated for 15 min at 37°C to induce cellular adhesion to Matrigel. Then, 20 µM OA and DMSO equivalent volume as control were ready to add to cells. After 6 h, representative images were taken using a Paula Nuc microscope (Leica Microsystems, Wetzlar, Germany). Images were processed and measured by ImageJ software. In this software, “Angiogenesis Analyzer” plugin (38) was used to analyze key neo‐angiogenesis markers: number of isolated segments, total length of isolated branches, number of master segments, number of meshes, number of nodes, number of segments, number of master junctions, total length of branches, and total length. The data presented are the data gathered from four C‐HUVEC strains and four GD‐HUVEC strains.

### Wound healing scratch assay

C‐ and GD‐HUVECs were grown in 24‐well plates coated with 1.5% gelatin until they reached 100% confluence in HUVEC complete medium. At this point, a serum‐starvation period was performed in 1% FBS HUVEC serum starvation medium for 24 h. Cells were scratched using a sterile p‐40 µL pipette tip and then the resulting wounds were gently washed with free‐FBS DMEM low glucose to remove released cells. Treatments were performed in the plates by adding DMSO and 20 µM OA in 0.5% FBS media. Additionally, 20% FBS was added as a positive control. After 12 h, the assay was stopped by fixing the cells with 4% formaldehyde (Applichem GmbH, Darmstadt, Germany) in PBS (Biowest, Nuaillé, France) for 10 min. Finally, cells were washed twice with PBS. Pictures were taken at 10× magnification using an optical microscope equipped with a digital camera (Motic Optic AE31, Motic Spain, Barcelona, Spain). Areas in the wounds at 0 h and 12 h were measured by ImageJ software. The initial cell area (0 h) was subtracted from the final cell area (12 h) and plotted in a graph as migration percentage (39).

### Focal adhesion quantification assay

C‐ and GD‐HUVECs were grown on round‐glass coverslips coated with 1.5% gelatin until they were sub‐confluent (60%) in HUVEC complete medium. At this time, cells were washed with serum‐deprived medium and then treated with 20 µM OA and DMSO equivalent volume (basal) in 0.1% FBS HUVEC starvation medium. After 24-h incubation, coverslips were fixed with 4% formaldehyde (Applichem GmbH, Darmstadt, Germany) in PBS (Biowest, Nuaillé, France) for 10 min and washed twice with PBS. Then, cells were permeabilized with 0.3% Triton X-100 (Sigma-Aldrich, St Louis, MO, USA) in PBS for 10 min. For immunostaining, a 30-min blocking was performed in PBS solution with 10% FBS, 5% skim milk (Beckton Dickinson, Franklin Lakes, NJ, USA), 0.3% bovine serum albumin (BSA, Sigma‐Aldrich, St Louis, MO, USA) and 0.1% Triton X-100. Subsequently, cells were incubated for 1 h with anti‐paxillin antibody, diluted in the above‐mentioned blocking solution without skim milk. Proper fluorescent‐labeled secondary antibodies (see the *Antibodies* section) were co‐incubated for 30 min with Alexa Fluor 594 conjugated phalloidin (Molecular Probes, Thermo Fisher Scientific, Waltham, MA, USA) and Hoechst 33258 (Fluka, Biochemika, Sigma‐Aldrich, St Louis, MO, USA) to reveal actin cytoskeleton and nuclei, respectively. Once the immunostaining was completed, representative pictures were acquired with a confocal microscope at 40x magnification (LSM 510 META from ZEISS, Jena, Germany). The setting of images was performed using Zeiss Efficient Navigation (ZEN) interface software (ZEISS, Jena, Germany). The “Z stack” ZEN tool was used in order to observe deep cytoskeleton structures (paxillin), taking picture slices along the *Z* axis. Then, picture slices were merged by the “Maximum intensity projection” ZEN tool. FA quantification was carried out as previously described by using CLAHE and Log3D macros for ImageJ (40). Essentially, FAs were quantified from paxillin-stained acquired pictures. We used four different replicates for each condition. Specifically, cell filopodia were selected as regions of interest (ROIs) and the resulting areas (containing FAs) were considered for further analysis. A number of five filopodia were considered from each picture. Then, the number of FAs were calculated in each filopodia by using the previously mentioned macros. The obtained number was divided by the total filopodia area to determine FA density in the cells.

### Antibodies

The following commercial primary antibodies were used: 1:1,000 anti‐phospho‐NF‐κB (Cell Signaling Technology, Danvers, MA, USA); 1:1,000 anti‐NF‐kB and 1:1,000 anti‐VCAM1 (Abcam, Cambridge, United Kingdom); 1:200 anti‐paxillin (Santa Cruz Biotechnology, Heidelberg, Germany); and 1:4,000 anti‐β‐actin (Sigma‐Aldrich, St Louis, MO, USA). Secondary antibodies were as follows: 1:1,000 anti‐rabbit IgG Horseradish peroxidase linked F(ab’)2 I fragment (from donkey) (GE Healthcare, GE, Little Chalfont, United Kingdom); 1:3,000 anti‐mouse IgG1 (BD Pharmingen, Beckton Dickinson, Franklin Lakes, NJ, USA); and 1:400 Alexa Fluor 488 conjugated anti‐mouse (from donkey) (Thermo Fisher Scientific, Rockford, IL, USA).

### Statistical analysis

The gathered data were represented and analyzed using GraphPad Prism v7 software. Classical statistical parameters were calculated and statistical tests were performed with a 95% confidence interval. Consequently, in each test, *p*‐values lower than 0.05 were considered to be statistically significant. At the figure legends, asterisks indicate statistically significant differences between assay conditions (*p < 0.05, **p < 0.005, ***p < 0.001, and ****p < 0.0001). Data were analyzed by a one‐way ANOVA test, comparing the mean of each condition with the mean of every other condition. Subsequently, a Tukey’s multiple comparisons test was performed. *p*‐values lower than 0.05 indicate statistically significant differences between the means of conditions.

## Results

### Oleanolic acid attenuates adhesion molecule overexpression induced by TNF‐α in C‐ and GD‐HUVEC

**Figure 1:**
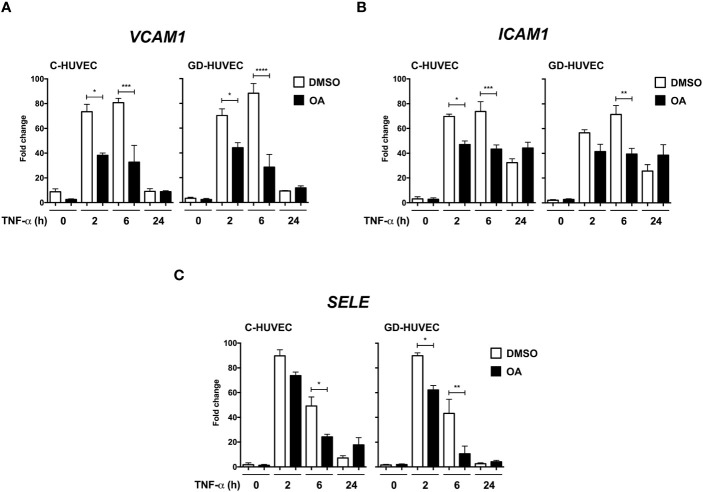
Oleanolic acid reduces the expression of adhesion molecule genes induced by TNF‐α. Gene expression analysis of **(A)** *VCAM‐1*, **(B)** *ICAM‐1*, and **(C)** *SELE* in C‐ and GD‐HUVECs pre‐treated 24 h with 20 µM OA (black) or DMSO equivalent volume (white, DMSO). After pre‐treatments, cells were stimulated with TNF‐α at 2, 6, and 24 h. Histograms represent mRNA relative expression of each gene (normalized with GAPDH expression) for both C‐ and GD‐HUVECs. Each condition represents the mean ± SEM using four different strains for C‐HUVECs and four other different strains for GD‐HUVECs. Asterisks indicate statistically significant differences between the selected conditions according to a one‐way ANOVA statistical analysis (*p < 0.05, **p < 0.005, ***p < 0.001 and ****p < 0.0001).

Adhesion molecule expression on endothelial cell surface is needed for immune cell recruitment to endothelial surface and, finally, migration to the inflammation source at the wound (41). However, an uncontrolled recruitment triggers endothelium dysfunction (42). Adhesion molecule gene expression, which is upregulated in endothelial cells in response to the pro‐inflammatory cytokine TNF‐α, was tested on HUVECs. Both C‐ and GD‐HUVECs were treated with 20 µM OA and then TNF‐α stimulated for the indicated times. On the whole, no gene expression differences were detected after 24 h pre‐treatment with OA (**Supplementary Figure 1**) (0 h). Generally speaking, adhesion molecule expression in untreated C‐ and GD‐HUVECs showed a strong response by TNF‐α at 2 and 6 h in all genes tested. However, beginning with the *VCAM1* gene (**Figure 1A**), a patent attenuation with OA was detected at 2 and 6 h in both C‐ and GD‐HUVECs. Strikingly, this reduction was even more significant in GD‐HUVECs at 6 h. Regarding *ICAM1* (**Figure 1B**), this OA-dependent decrease was less patent but remained significant, mostly at 6 h, in C‐ and GD‐HUVECs. The expression of the third adhesion molecule tested *SELE* (**Figure 1C**) also showed a strong attenuation with OA, which was more patent in the GD phenotype. At 24 h, in both types of endothelial cells and both conditions, all adhesion molecules showed a drop in their expression; therefore, we could not see any statistically significant differences.

**Figure 2:**
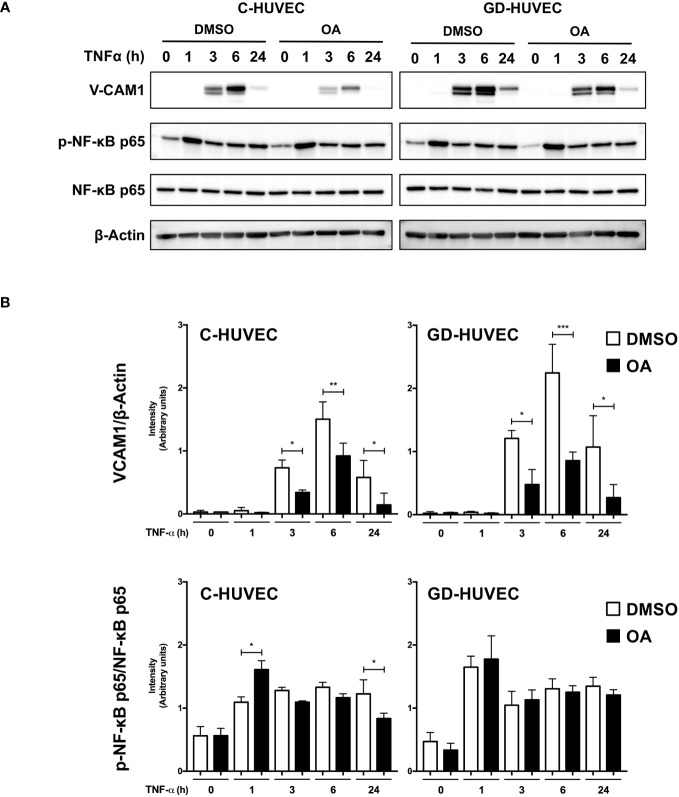
Oleanolic acid reduces total VCAM1 protein expression in C‐ and GD‐HUVECs induced by TNF‐α. **(A)** Total protein extracts from sub-confluent C‐ and GD‐HUVECs pre‐treated with 20 µM OA or DMSO equivalent volume, and then stimulated with TNF‐α at 1, 3, 6, and 24 h. These extracts were assayed at these times targeting the following: VCAM1, phospho‐NF‐κB, and NF‐κB. β‐Actin was used as a loading control. A representative experiment is shown. **(B)** Column bar graphs represent intensity values of each protein assayed by Western blot, by collecting the data of four C‐HUVEC and four GD‐HUVEC strains. Intensity values were quantified and gathered by ImageJ software. Asterisks indicate statistically significant differences between the selected conditions according to a one‐way ANOVA statistical analysis: (*p < 0.05, **p < 0.005, and ***p < 0.001).

Subsequently, we studied VCAM1 total protein amount in C‐ and GD‐HUVECs using the same experimental design (**Figure 2**). At basal conditions, total VCAM1 protein levels were detected in both cell lines after 3 h of TNF‐α stimulation, showing its highest level at 6 h, whereas 24 h later, the levels plummeted. To begin with, GD‐HUVECs showed higher protein levels than control ones. Interestingly, C‐ and GD‐HUVEC lysates with OA pre‐treatment showed significantly lower total VCAM1 protein levels than control, which suggested a strong OA attenuation on the TNF‐α stimulation. Endothelial cell stimulation with TNF‐α induces the expression of adhesion molecules through the participation of nuclear factor‐κB (NF‐κB) that is phosphorylated at Ser 536 and then translocates to the nucleus where it activates the expression of *VCAM1*, among others (43–45). However, when Ser 536 phosphorylated NF‐κB was assayed in response to TNF‐α stimulation, no significant differences were found between 20 µM OA treated and non-treated C‐ or GD‐HUVECs. Only a slight decrease of phospho‐NF‐κB level was noticed in C‐HUVECs after 24 h OA stimulation and only in the TNF‐α sample.

All these data suggest that OA attenuates the expression of VCAM1 protein and of the *VCAM1, SELE*, and *ICAM1* genes in response to TNF‐α in HUVECs regardless of its glucose-affected condition.

### Oleanolic acid reduces the number of monocytes adhered to GD‐HUVECs

**Figure 3:**
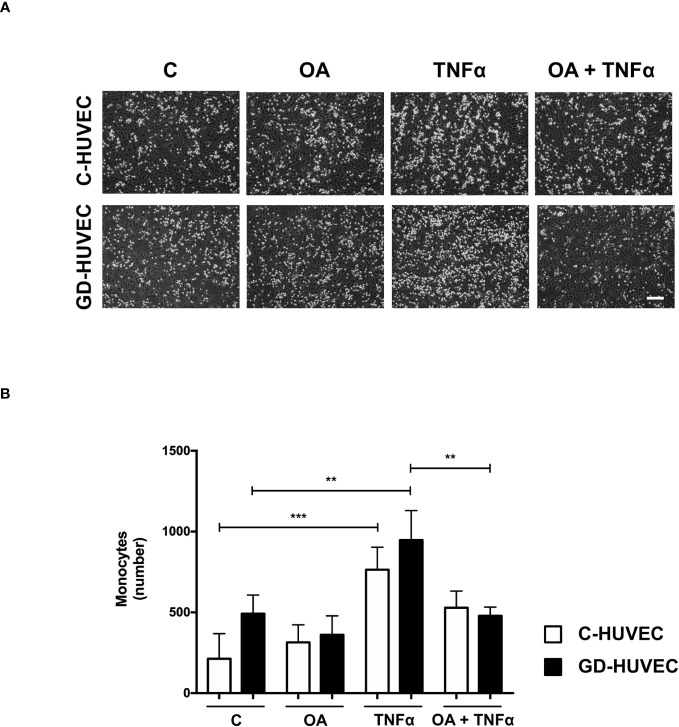
Oleanolic acid reduces the number of monocytes adhered to C‐ and GD‐HUVECs. **(A)** For monocyte adhesion experiment, C‐ and GD‐HUVEC monolayers were left untreated unless otherwise indicated (OA), where they were pre-treated with 20 µM OA for 24 h. Subsequently, TNF‐α was added for 16 h to either untreated or OA pre‐treated HUVECs (TNF‐α and OA+TNF‐α conditions). At this point, monocytes were added and the experiment was completed. Representative pictures of C‐ and GD‐HUVECs are shown for each condition. **(B)** Graph represents the number of adhered U937 monocytes in each field of 12 fields. Each condition represents the mean ± SEM obtained from the data collection of four different strains for both C‐HUVECs and GD‐HUVECs. Asterisks indicate statistically significant differences between the selected conditions according to a one‐way ANOVA statistical analysis (*p < 0.05, **p < 0.001). The scale bar indicates 100 µm.

Given the attenuation effect of OA on adhesion molecule expression, a monocyte adhesion assay was conducted on C‐ and GD‐HUVEC monolayers (**Figure 3**). HUVECs pre-treated or not with OA were subjected to 16 h TNF‐α, to study whether the OA effect on adhesion molecules correlated with the number of monocytes adhered to their surface. When C‐ and GD‐HUVECs were stimulated with TNF‐α, there was a clear increase in the number of adhered monocytes, which was slightly higher on GD‐HUVECs (**Figure 3**). Interestingly, OA pre‐treatment decreased the number of monocytes in both C- and GD‐HUVECs (**Figure 3**) before and after treatment with TNF‐α.

Overall, this functional assay reveals less monocyte–endothelial interaction triggered by TNF‐α in both C‐ and GD‐HUVECs when the cells are previously treated with OA.

### Oleanolic acid improves neo‐angiogenesis in GD‐HUVECs

**Figure 4:**
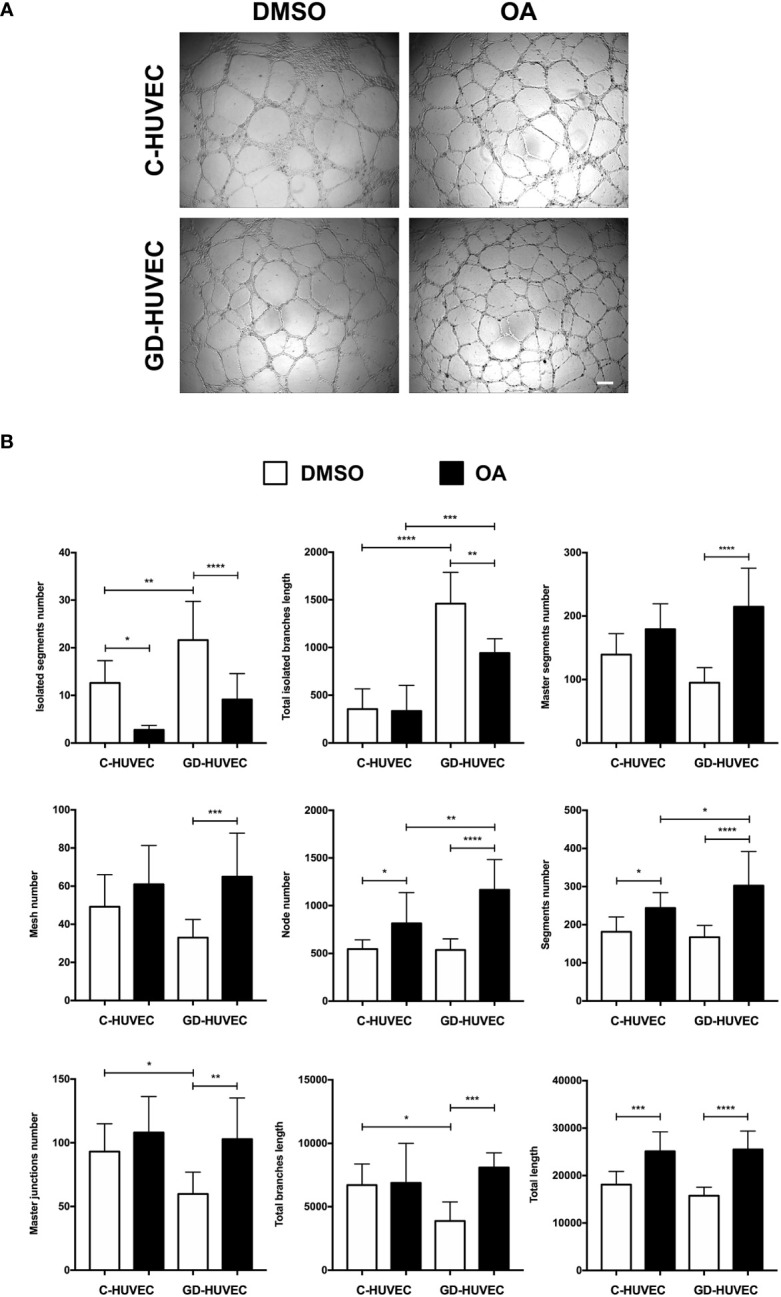
Effect of OA on tube-like structure formation capacity on Matrigel. Tube‐like structure formation ability on Matrigel after 6-h treatment with 20 µM OA; a DMSO equivalent volume was added as control condition. **(A)** Representative pictures of C‐ and GD‐HUVECs for both experimental conditions. Scale bar indicates 200 µm. **(B)** Graphs representing multiple angiogenic parameters analyzed: number of isolated segments, total length of isolated branches, number of master segments, number of meshes, number of nodes, number of segments, number of master junctions, total length of branches, and total length. Each bar in the plot represents the mean ± SEM using three different strains for C‐HUVECs and also three for GD‐HUVECs. Asterisks indicate statistically significant differences between the selected conditions according to a one‐way ANOVA statistical analysis (*p < 0.05, **p < 0.005, ***p < 0.001, and ****p < 0.0001).

Matrigel tube formation assay with HUVEC is a well‐established and informative test to evaluate the angiogenesis function of endothelial cells *in vitro* (28, 38, 46). C‐ and GD‐HUVECs were seeded in Matrigel and treated with OA for 6 h. Representative pictures indicated a greater network complexity in both HUVEC phenotypes under OA conditions (**Figure 4A**); however, the possible effects of OA on the GD-HUVEC versus the C-HUVEC were difficult to interpret. To gain more knowledge about angiogenesis features with OA, nine key parameters related to this meshed network were measured (**Figure 4B**). The number of isolated segments were higher in GD- compared to C-HUVEC, as it was reduced with OA. Similarly, total length of isolated branches was reduced with OA in GD-HUVEC. In addition, the number of master segments, which were affected in GD-HUVEC, was ameliorated with OA. Finally, both the number of master junctions and total length of branches were deficient in GD-HUVEC when compared to C-HUVEC. In all cases, the presence of OA produced and improvement of the parameters. Finally, the number of nodes, the number of segments and total length, all representing the complexity of the network, were all increased by OA in both C- and GD-HUVEC, but showed a more powerful effect on GD‐HUVEC.

Altogether, GD-HUVEC exhibited poor performance for most of the measured tube‐formation parameters. Generally, the quantification of all these parameters suggested that OA generally improved angiogenesis, with clear healing tendencies for the GD‐HUVECs.

### Oleanolic acid enhances C‐ and GD‐HUVEC migration

**Figure 5:**
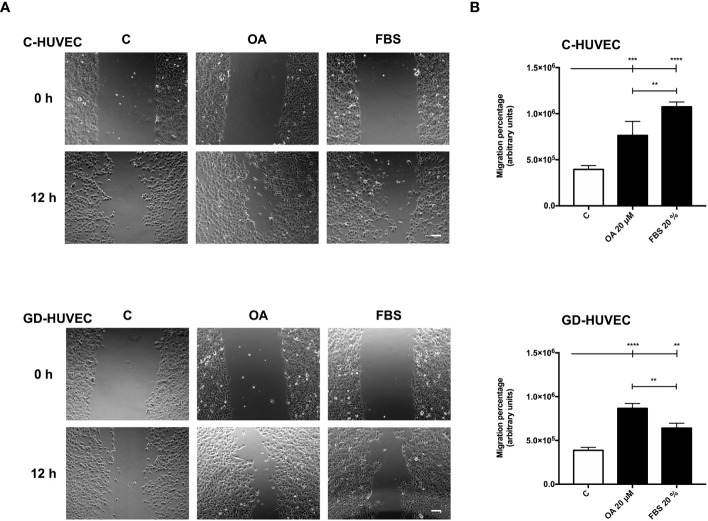
Oleanolic acid induces C‐ and GD‐HUVEC migration in wound healing scratch assays. Confluent C‐ and GD‐HUVECs were scratched with a pipette tip and allowed to migrate for 12 h. **(A)** Representative images of the wound healing assay with cell migration under basal conditions (control, C), compared to those with 20 µM OA after 24-h treatment. A condition with 20% FBS was added as positive‐migration control. Scale bar indicates 200 µm. **(B)** Graphs represent C‐ and GD‐HUVEC migration as the difference between areas at 0 h and 12 h in each condition, named as migration percentage. Asterisks indicate statistically significant differences between conditions according to a one‐way ANOVA statistical analysis (**p < 0.005, ***p < 0.001, and ****p < 0.0001).

Cell migration, a crucial process in wound healing to restore skin integrity, is enhanced by OA in epithelial cells (7, 8). Interestingly enough, migration also contributes to the organization and formation of new vessels (47). Therefore, we performed scratch assays on confluent C‐ and GD‐HUVECs to see whether OA could also have this effect on endothelial cells. C‐ and GD‐HUVECs were scratched and allowed to migrate for 12 h in the presence of OA (**Figure 5A**). Strikingly, OA activity promoted the migration of both C‐ and GD‐HUVECs from the wound edges, since the wound gap area was surrounded with endothelial cells. To better comprehend the level of this promoting effect of OA, cell migration was quantified measuring the resulting areas of the wounds (**Figure 5B**). Thus, the obtained migration percentages in both C‐ and GD‐HUVECs were clearly significant between basal condition and OA. Interestingly, 20% FBS was used as a positive control of cell migration, but the resulting migration with GD‐HUVEC under this condition was significantly lower than with OA.

Given these results with wound healing scratch assays on HUVECs, OA showed cell migration promoting effects on endothelial cells that could probably enhance the wound healing process together with neo‐angiogenesis.

### Oleanolic acid increases focal adhesion number in C‐ and GD‐HUVECs and their dynamization

**Figure 6:**
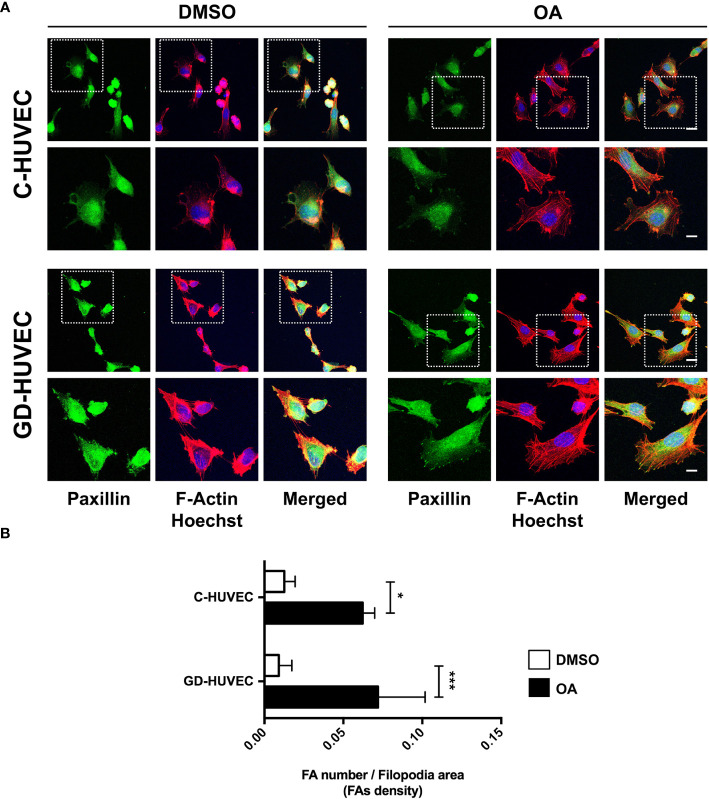
Oleanolic acid triggers focal adhesion remodeling in C‐ and GD‐HUVECs revealed by paxillin. **(A)** Sub‐confluent C‐ and GD‐HUVECs were treated for 24 h with 20 µM OA and DMSO equivalent volume. Cells were immunostained with specific antibodies against paxillin. Co‐staining with phalloidin and Hoechst‐33258 was used to show actin cytoskeleton and nuclei, respectively. Paxillin: green. Actin fibers (F‐Actin): red. Nuclei: blue. Images obtained with a confocal microscope at 63× magnification and their corresponding insets for a detailed view of paxillin structures. This experiment was repeated at least three times. 63× picture scale bar indicates 25 µm. Inset scale bar indicates 5 µm. **(B)** Column bar graphs show the quantification of the density of FAs (as FA number per filopodia area). Asterisks indicate statistically significant differences between conditions according to a one‐way ANOVA statistical analysis (*p < 0.05 and ***p < 0.001).

It is known that OA‐triggered molecular effects on cell migration include the role of cell architecture, by dynamizing actin cytoskeleton and FA remodeling (9, 48, 49). We performed immunocytochemistry assays in sub‐confluent C‐ and GD‐HUVECs targeting actin fibbers (F‐actin) and paxillin to reveal FAs. With regard to cell morphology, untreated C‐ and GD‐HUVECs exhibited a morphology related to a stressed condition due to the low amount of FBS (0.1%), with no apparent FA‐like structures (**Figure 6A**). By contrast, C‐ and GD‐HUVECs treated with OA displayed filopodia and lamellipodia garnished with FAs. Actin fibers were also modified by OA presence, because they were encompassing the newly formed filopodia and lamellipodia in response to OA. Interestingly and in line with this, the quantified FA density, revealed by paxillin staining, exhibited a significant increase in OA‐treated C‐ and GD‐HUVECs versus untreated ones (**Figure 6B**). This increase was even more significant in GD‐HUVECs than in C‐HUVECs.

Actin fiber and FA data revealed that HUVECs changed their cell architecture during OA‐stimulated cell migration, thus suggesting a high dynamization of the migration-related machinery in both C- and GD-HUVECs.

## Discussion

The results of this study provide intriguing insights into the effects of OA treatment on C- and GD-HUVECs in the context of inflammation and angiogenesis.

Plant‐derived bioactive compounds present in various dietary sources have been widely studied for their significant effects to rescue endothelial cell function (50–52). Their antioxidant, anti‐inflammatory, vasodilatory, angiogenic, and protective properties collectively contribute to the preservation of vascular health and the prevention of endothelial dysfunction‐associated diseases (53–55). Indeed, a large number of these bioactive molecules or peptides modulate the signaling pathway of nuclear factor‐kappa B (NF‐κB), which is needed for adhesion molecules and pro‐inflammatory cytokine expression (43, 56). For instance, studies have shown that carotenoids lycopene and β‐carotene have anti‐inflammatory effects on both C‐ and GD‐HUVECs (31, 33). Indeed, the addition of these carotenoids under TNF‐α stimulation show less monocyte–endothelial cell interaction, enhanced by less ICAM1 and VCAM1 membrane exposure and total expression. All these effects depend on the attenuation that these carotenoids have on NF‐κB phosphorylation and translocation to the cell nucleus (31, 33). In fact, the effects of pentacyclic triterpenes, OA, and its isomers ursolic acid (UA) and maslinic acid (MA) are similar to carotenoids and have been addressed *in vitro* by using regular HUVEC phenotype. Thus, these studies showed attenuation effects on adhesion molecule expression under inflammation conditions (29, 30, 57). However, there are no studies so far on the effects of OA on hyperglycemia‐modified cells (GD‐HUVECs), which have remarkably impaired functionality. Moreover, the concentration of serum used in those assays was not always clarified, a factor that is critical to properly study OA effects *in vitro*, since serum proteins buffer OA activity and modify its optimal concentration of use (7, 8). Indeed, OA effects and bioavailability depend on the final OA concentration, the cell type used, and the serum concentration. High concentrations of OA produce cytotoxic and antiproliferative effects, while low concentrations do not produce any beneficial effect on cells (8, 58, 59). This is the reason why, in the present study, an MTT assay was conducted with C‐ and GD‐HUVECs under the lowest possible serum concentration (0.5% FBS). In this way, a 20 µM OA concentration was established seeking a compromise between the optimal effects of OA and the abolition of the serum buffer effect, together with cell viability compatibility.

Furthermore, it should be noted that OA treatments, followed by TNF‐α induction, should be performed at longer incubation times to unravel OA ameliorative effects on inflammation (30). Indeed, the highest expression of adhesion molecules *ICAM1*, *VCAM1*, and *SELE* in C‐ and GD‐HUVECs was detected at 2 h and 6 h after TNF‐α addition, thus showing a clear inflammatory profile. Strikingly, pre‐treating the same endothelial cells with OA before TNF‐α clearly attenuated adhesion molecule overexpression by the cytokine, especially on *VCAM1* and *SELE*. In addition to this, the preventive effect produced by OA was even more patent in GD‐HUVECs, probably because of their senescent phenotype and endothelial dysfunction (26). Interestingly enough, in the case of *ICAM1*, at 24 h, gene expression levels were not fully abrogated by OA in C‐ and GD‐HUVECs. This could be explained by other functions of this integrin, since controlled levels of ICAM1 on the cell surface are needed during wound healing to promote endothelial cell migration, thus leading to neo‐angiogenesis (60). In particular, *VCAM1* showed the strongest attenuation by OA in C‐ and GD‐HUVECs, and also showed decreased protein amount. However, we saw a window transient effect of TNF‐α, since VCAM1 levels decreased at 24 h. Despite this, we still observed the mitigating effect of OA on VCAM1 protein levels. It should be highlighted that *in vitro* assays have this limitation, because, in a tissue with chronic inflammation, we would see sustained high levels of VCAM1 and other adhesion molecules due to the constant production of TNF‐α and other pro‐inflammatory cytokines (61). VCAM1 is endothelium‐specific and this TNF‐α inducible molecule is necessary for monocyte extravasation (44). NF‐κB activation by its phosphorylation on the p65 subunit is required to promote adhesion molecule expression in the cell nucleus, such as *VCAM1*, among others (43). Although we observed lower VCAM1 protein levels with OA pre‐treatment, we did not observe any differences on phospho-NF‐κB p65. In contrast, in a similar set of experiments, the precondition of HUVECs with amniotic membrane was able to reduce the levels of phosphorylation of phospho‐Ser‐536 NF‐κB p65 in response to TNF‐α, which was coherent with an attenuation of the NF‐κB p65 nuclear translocation and a reduction of the expression of *VCAM1* (28). Thus, our results have to be explained by the fact that the OA attenuation effect on VCAM1 could be due to different molecular mechanisms or also to the way it is synthesized. In fact, it has been shown that UA, an OA isomer, blocks VCAM1 traffic to the membrane (62). Another possibility could be that the amount of anchored‐membrane VCAM1 is regulated by proteases, where specifically TNF‐α converting enzyme (TACE/ADAM17) proteolyzes this molecule and releases it to the extracellular medium (44). Therefore, OA could be enhancing TACE/ADAM17 protease activity on VCAM1, thus decreasing its protein levels in endothelial cells. However, a complicated regulation must be involved, since the expression of *VCAM1* is effectively attenuated by the presence of OA. A more plausible, although uncertain, mechanism of regulation could be related to something different from NF‐κB transcription factor or even its phosphorylation at Ser 536 residue. Further research is necessary to better clarify the mechanism behind *VCAM1* regulation in this context.

An excessive release of TNF‐α in chronic inflammation conditions produces the overexpression of adhesion molecules on the endothelium surface. As a consequence, the uncontrolled adhesion and transmigration of immune cells occur, thus triggering endothelial cell apoptosis (42). E‐selectin acts at the first steps of monocyte recruiting to produce their tethering and rolling (63). Then, integrins ICAM1 and VCAM1 secure the adhesion and allow monocyte extravasation to the injured wound (64). In a chronic wound, the high recruitment of monocytes leads to an uncontrolled population of M1 macrophages in the wound, which have hyperinflammatory, reduced phagocytic activity, and increase oxidative stress (14). By contrast, a regular recruitment of monocyte population swings toward M2 macrophages, with anti‐inflammatory, regenerative, and tissue remodeling properties, all in line with a healing wound (14). OA effects regarding monocyte adhesion are strongly coherent with the observed changes of adhesion molecule levels. In the context of either chronic or diabetic wounds, OA-reduced levels of VCAM1 on endothelial cells surely imply a better inflammation resolution.

Diabetes negatively affects angiogenesis (22, 24). Considering tube formation assays, GD‐HUVEC exhibited a poorer tube formation; for instance, the number and length of isolated segments in basal condition was higher in GD‐HUVEC. Strikingly, OA treatment clearly ameliorated this impairment in GD‐HUVECs and, although more lightly, also in C‐HUVECs. Coherently, positive features such as the number of master segments, the number of meshes, and the length of branches were significantly improved by OA only in GD‐HUVECs. Indeed, this could be explained by the GD phenotype, which, in contrast to C‐HUVEC, showed poorer performance for these parameters in basal conditions. This behaviour strongly suggests that OA restores GD‐HUVEC to a more regular angiogenic phenotype, but does not intrinsically affect C‐HUVEC’s capability of achieving a full network. Overall, the network complexities achieved for both types of cells were higher with OA, as reflected by the observed incremented number of nodes, branches, master junctions, and the total length of the networks. These changes indicate that OA enhances all aspects of the complexity of the vascular network, which may have a positive impact on tissue regeneration in a complex healing wound milieu. Nonetheless, a good line of research could be testing the effects of OA on more complex systems, because tube formation assays on Matrigel do not compile/integrate endothelial cell interaction with other cell types, as happens during neo‐angiogenesis in a real wound. Therefore, a 3D co‐culture of endothelial cells with both primary fibroblasts and keratinocytes, which exhibit features more similar to natural skin, could be considered a good option to further assess the effects of OA on wound healing (65). Moreover, there are well‐established *in vivo* angiogenesis assays that can unravel potential OA effects; for instance, one of the best is chorionallantoic membrane assays in chicken embryo, which are widely used in vascular biology (66). Regarding the potential molecular effects behind OA angiogenesis promotion, we would like to conduct future experiments in order to study the effects of OA on the stimulation of VEGFR‐2, given its importance in angiogenesis (15, 67, 68), and due to the fact that OA has been directly involved in the activation of the similar function and structure receptor: EGFR (7, 8).

Cell migration is carried out by endothelial cells together with proliferation to enhance angiogenesis and vasculogenesis (69). According to the data of the scratch assays, OA was also capable of enhancing this process in both C‐ and GD‐HUVECs to the same extent. However, FBS stimulation was unable to match the levels achieved by the OA stimulation for the GD phenotype. These data indicate that OA, but not FBS, rescues, in GD‐HUVEC, an impaired migration mechanism resistant to the serum rescue. Thus, OA may trigger a particular molecular mechanism that is revealed only in the GD‐HUVEC‐impaired cells. Indeed, the quantification of FA density upon treatment with OA, detected by paxillin immunostaining, was stronger in GD‐HUVECs than in C‐HUVECs. Overall, the collected data of FAs strongly suggest that, generally, OA promotes a better endothelial cell movement to manage migration in both GD- and C‐HUVECs. OA also contributes positively to angiogenesis by cell migration promotion, which is a favorable condition for tissue repair in a wound healing context (69).

Our findings suggest a clear OA ability to rescue the altered features of an endothelium affected by high blood sugar levels, which correlate with impaired metabolism and inflammation (25, 42, 70). It is well-known that, in order to ameliorate these processes, signaling pathways depending on the activation of G protein‐coupled receptors (GPCRs) take place to protect cells from injury and malfunction. Concretely, the Takeda G protein‐coupled receptor (TGR5), also known as Gpbbar1, is a transmembrane‐type bile acid receptor that has been found to regulate a large number of specific molecular pathways (71, 72). Interestingly, TGR5 modulates inflammation by decreasing adhesion molecule expression in endothelial cells and blocking pro‐inflammatory cytokines released by immune cells (71). Moreover, this receptor is also linked to tyrosine kinase receptor (RTK) transactivation by second messengers (73, 74). Strikingly, some evidence points out the interaction between OA, which has a similar chemical structure to bile acids, and TGR5, with OA behaving as a clear agonist of TGR5 (75). For these reasons, it is remarkable to suggest that probably all OA promotion and modulation effects related to monocyte adhesion, angiogenesis, and cell migration on C‐ and GD‐HUVECs could be related to the interaction between TGR5 and OA. Thus, more research is needed to decipher this molecular mechanism, which may solve some of the conundrums revealed in our data. In addition, other mechanisms may be involved under OA effects regarding regulatory non‐coding RNA expression, as microRNAs (miRs) have recently gained prominence due to their role in regulating several essential processes in endothelial cells (76, 77). For instance, it is shown that miR‐4432 controls the expression of fibroblast growth factor binding protein 1 (FGFBP1), which is needed to preserve endothelial barrier function in the brain (78). On top of that, other studies reported that low expression levels of miR‐145 and miR‐885 cause thrombotic risk and mortality in COVID‐19 patients; thus, the expression of these miRs in endothelial cells is critical to prevent a prothrombotic condition during the infection (79). Therefore, further studies focusing on OA’s contribution to these miR expressions in endothelial cells seem very pertinent.

**Figure 7:**
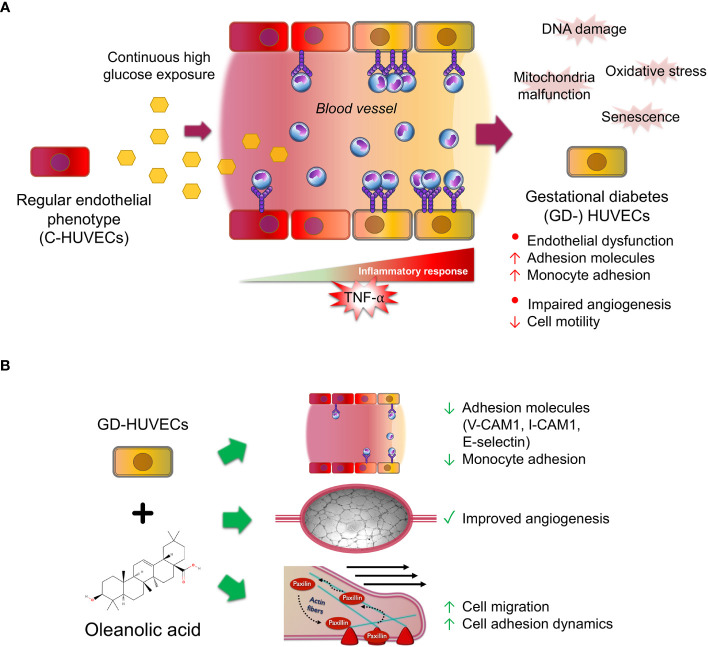
Oleanolic acid rescues multi‐affected GD‐HUVEC features caused by high blood glucose levels. **(A)** When regular endothelial cells (C-HUVECs) are exposed to a continuous high glucose exposure in blood vessels, several changes on their phenotype occur. These cells undergo the effects of high oxidative stress and damages on their DNA and mitochondria malfunction, thus triggering cellular senescence. As a result, these cells have an excessive and uncontrolled inflammation response when stimulated by pro‐inflammatory cytokines such as TNF‐α, triggering high adhesion molecule exposure on endothelium surface, subsequently displaying a high recruitment of circulating monocytes, and resulting in endothelial dysfunction. Moreover, these cells have an aberrant and limp tube formation (angiogenesis). **(B)** Strikingly, OA pre‐treatment in GD‐HUVECs before TNF‐α addition attenuates key adhesion molecule overexpression of VCAM1, ICAM1, and E-selectin, resulting in less adhesion of the monocytes. Furthermore, OA displayed promotion effects on GD-HUVECs by restoring the impaired angiogenesis. Moreover, cell migration, a crucial process for angiogenesis, is also promoted by OA because it increases endothelial cell migration and adhesion dynamics by focal adhesion formation.

To sum up, this study sheds some light on the multifaceted effects of OA on inflammation, angiogenesis, and migration in C‐ and GD‐HUVECs (**Figure 7**). The findings underline the potential of OA as a therapeutic agent for restoring vascular function and ameliorating inflammation excess in diabetic wounds. However, further research is needed to unravel the precise underlying molecular mechanisms driving these effects in order to evaluate the translational potential of OA clinical treatments for the management of complex wounds.

## Data availability statement

The original contributions presented in the study are included in the article/**Supplementary Material**. Further inquiries can be directed to the corresponding authors.

## Ethics statement

The studies involving humans were approved by Institutional Committee on Human Experimentation (reference number 1879/09COET). The studies were conducted in accordance with the local legislation and institutional requirements. The participants provided their written informed consent to participate in this study.

## Author contributions

JS-F: Data curation, Formal Analysis, Investigation, Methodology, Writing – original draft, Writing – review & editing. IC: Data curation, Investigation, Methodology, Writing – review & editing. AP: Conceptualization, Data curation, Funding acquisition, Supervision, Validation, Writing – review & editing. JG: Funding acquisition, Project administration, Supervision, Writing – review & editing. CP: Conceptualization, Data curation, Investigation, Methodology, Supervision, Writing – review & editing. FN: Conceptualization, Funding acquisition, Investigation, Methodology, Project administration, Supervision, Validation, Writing – review & editing.
