# Supplementary figures and images for: Oleanolic acid rescues critical features of umbilical vein endothelial cells permanently affected by hyperglycemia
Source: Front Endocrinol (Lausanne). 2023 Dec 13;14:1308606. doi: 10.3389/fendo.2023.1308606 (PMC10773851; doi:10.3389/fendo.2023.1308606)

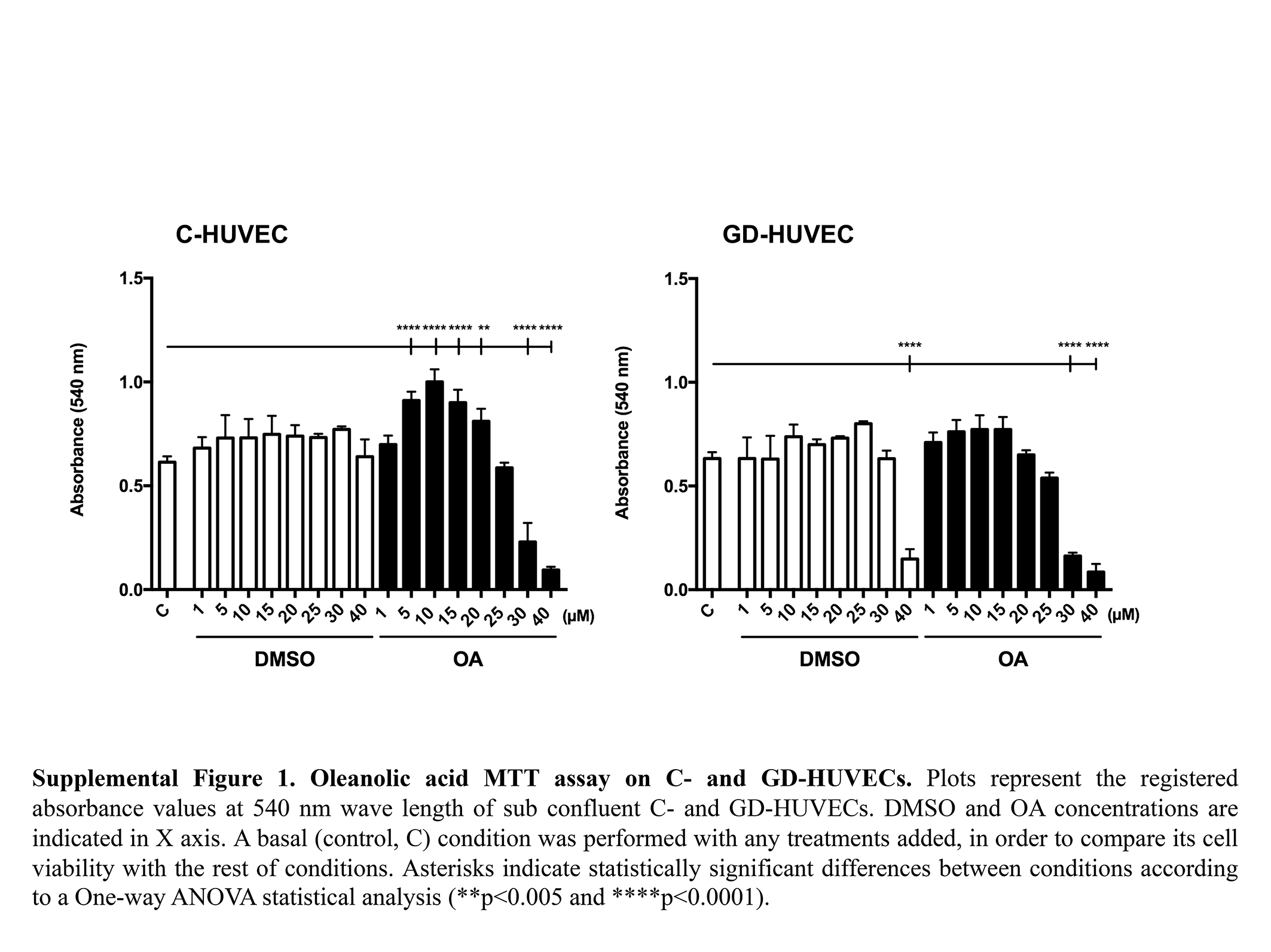

Supplement: Supplementary file 1 [file Image_1.tif]

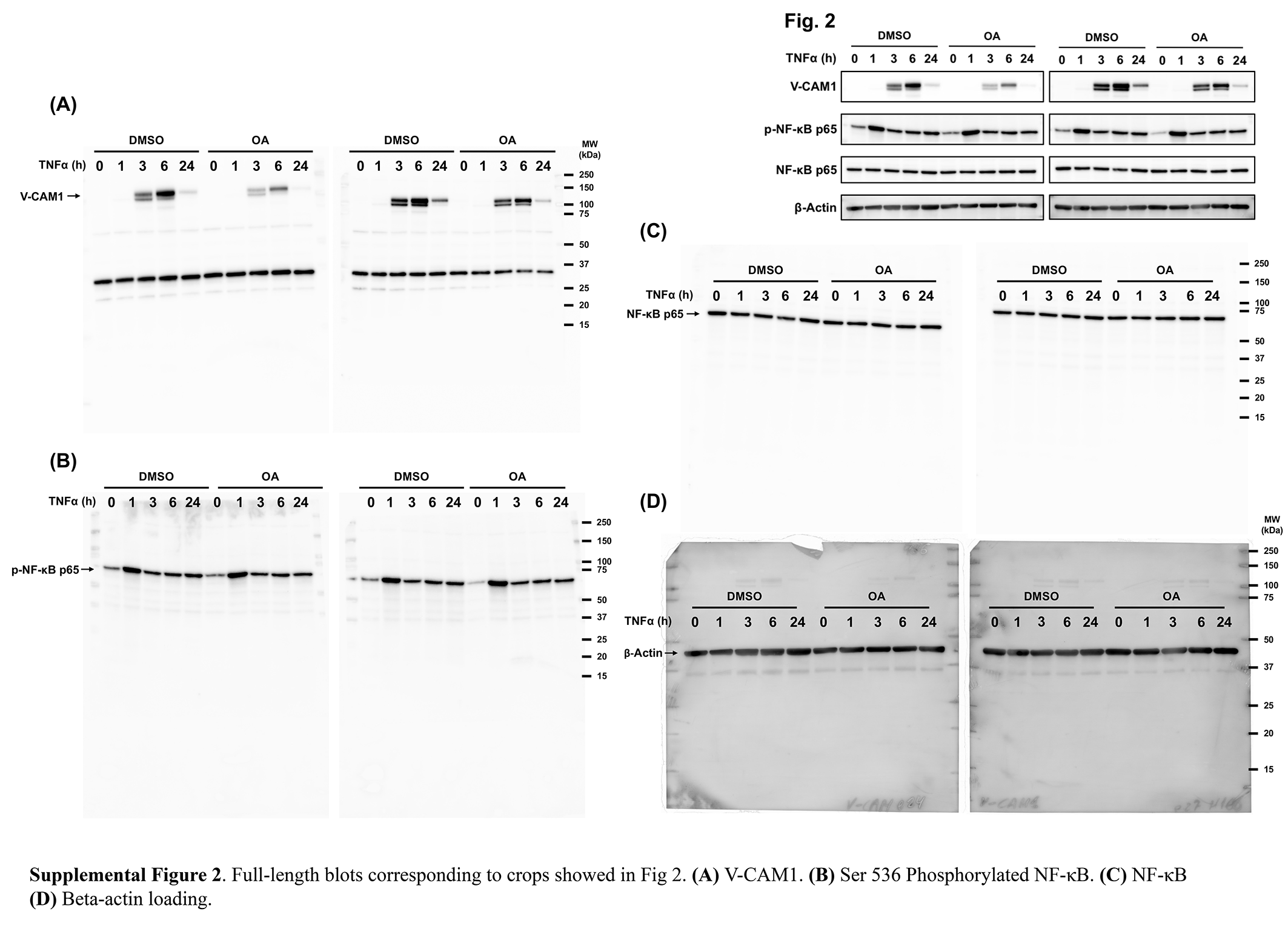

Supplement: Supplementary file 2 [file Image_2.tif]
